# Adenoidectomy for middle ear disease in cleft palate children: a systematic review

**DOI:** 10.1007/s00405-021-07035-6

**Published:** 2021-08-28

**Authors:** Cecilia Rosso, Antonio Mario Bulfamante, Carlotta Pipolo, Emanuela Fuccillo, Alberto Maccari, Paolo Lozza, Alberto Scotti, Antonia Pisani, Luca Castellani, Giuseppe De Donato, Maria Chiara Tavilla, Sara Maria Portaleone, Giovanni Felisati, Alberto Maria Saibene

**Affiliations:** Otolaryngology Unit, Department of Health Sciences, Santi Paolo E Carlo Hospital, Università Degli Studi Di Milano, Via Antonio di Rudinì, 8, 20142 Milan, Italy

**Keywords:** Otitis media, Hearing loss, Cleft palate, Cleft lip, Adenoids, Adenoidectomy

## Abstract

**Purpose:**

Cleft palate children have a higher incidence of otitis media with effusion, more frequent recurrent acute otitis media episodes, and worse conductive hearing losses than non-cleft children. Nevertheless, data on adenoidectomy for middle ear disease in this patient group are scarce, since many feared worsening of velopharyngeal insufficiency after the procedure. This review aims at collecting the available evidence on this subject, to frame possible further areas of research and interventions.

**Methods:**

A PRISMA-compliant systematic review was performed. Multiple databases were searched with criteria designed to include all studies focusing on the role of adenoidectomy in treating middle ear disease in cleft palate children. After duplicate removal, abstract and full-text selection, and quality assessment, we reviewed eligible articles for clinical indications and outcomes.

**Results:**

Among 321 unique citations, 3 studies published between 1964 and 1972 (2 case series and a retrospective cohort study) were deemed eligible, with 136 treated patients. The outcomes were positive in all three articles in terms of conductive hearing loss improvement, recurrent otitis media episodes reduction, and effusive otitis media resolution.

**Conclusion:**

Despite promising results, research on adenoidectomy in treating middle ear disease in the cleft population has stopped in the mid-Seventies. No data are, therefore, available on the role of modern conservative adenoidectomy techniques (endoscopic and/or partial) in this context. Prospective studies are required to define the role of adenoidectomy in cleft children, most interestingly in specific subgroups such as patients requiring re-tympanostomy, given their known risk of otologic sequelae.

**Supplementary Information:**

The online version contains supplementary material available at 10.1007/s00405-021-07035-6.

## Introduction

Children born with a nonsyndromic cleft palate, with or without cleft lip (CP ± L), have a higher incidence of otitis media with effusion (OME), more frequent recurrent acute otitis media (RAOM) episodes, and worse early age OME-related conductive hearing losses (ORCHL) than non-cleft children [1–3]. With a wide variability among studies and age groups, OME in CP ± L children has been shown to reach incidences as high as 90% in the first year and 97% within the first 2 years of life [4].

Studies suggest that both early tympanostomy and early surgical cleft repair lead to favorable audiological results in this population [5]. Nevertheless, the role of repeated tympanostomy has been shown to correlate (though with a possible consistent selection bias) with a higher prevalence of chronic otitis media [5] in a population already at a higher incidence of re-tympanostomy when compared to non-cleft children [6].

In the general pediatric population suffering from OME and RAOM, there is conspicuous, though often low-level, evidence for the role of adenoidectomy and/or tympanostomy for OME, ORCHL, and RAOM [7–9]. Most specifically, adenoidectomy has proved beneficial in treating OME in the pediatric population, while its role in hearing thresholds and RAOM episodes is less defined [7]. Analogously, the role of tympanostomy for ORCHL is unclear and limited in time [8], while it appears moderately beneficial in reducing RAOM episodes [9]. NICE guidelines, for example, include adenoidectomy as a treatment option for OME [10], while the Italian Pediatric Otolaryngology society guidelines recommend adenoidectomy in carefully selected cases of OME and RAOM, with adenoiditis or Eustachian tube obstruction [11].

Conversely, data on adenoidectomy for middle ear disease in CP ± L children are scarce at best. Many authors discouraged the use of adenoidectomy in this population fearing worsening of velopharyngeal insufficiency [12], though endoscopy-, mirror- and/or power-assisted modern techniques of adenoidectomy have proven safe also in this population [13].

This review aims at filling this knowledge gap by systematically collecting all the available evidence on the role of adenoidectomy in CP ± L in treating OME, RAOM, and ORCHL, to frame possible areas of further research and interventions. Furthermore, the discussion section also provides a narrative literature review on modern surgical approaches to adenoidectomy in CP ± L children, aimed at briefly depicting current surgical management options.

## Methods

This review protocol has been registered in the International Prospective Register of Systematic Reviews (PROSPERO) (ID CRD42021221115).

### Search strategy

A systematic review was conducted between Nov 1 and Dec 31, 2020, according to the Preferred Reporting Items for Systematic Reviews and Meta-analyses (PRISMA) reporting guidelines [13]. We conducted systematic electronic searches for studies in the English, Italian, German, French or Spanish language reporting original data obtained from humans focused on the role of adenoidectomy in the treatment of middle ear disease in the cleft (lip and) palate population, with no publication date restrictions.

On Nov 18th, 2020, we searched the MEDLINE, Embase, Web of Science, Cochrane Library, and ClinicalTrials.gov databases with the following extremely wide search string (common to all databases):

(“adenoidectomy” OR “adenotonsillectomy”) AND “cleft”.

We focused on studies on CP ± L children, diagnosed with OME, RAOM, and/or ORCHL, undergoing adenoidectomy or adenotonsillectomy. As per study design, we included only studies where the patients were diagnosed with (a) OME, defined with tympanometry or otoscopy; (b) ORCHL, quantified with pure tone audiometry; or (c) RAOM. Similarly, another inclusion criterion for studies was reporting respective outcomes as (a) resolution of OME, defined with normal tympanometry or otoscopy; (b) improvement of ORCHL, quantified with pure tone audiometry; or (c) reduction of acute otitis media episodes.

We excluded meta-analyses, systematic and narrative reviews, and case reports. There was no minimum study population size required. References from reviews and included articles were nevertheless checked for additional potentially relevant studies.

Abstracts and full texts were reviewed in duplicate (C. R. and A.M.B). All disagreements were resolved by evaluation from a third rater (A.M.S.).

### PICO criteria

The PICO criteria for the present review were as follows:

- Patients: Cleft palate or cleft lip and palate children with RAOM, ORCHL and/or OME
- Intervention: Adenoidectomy (with or without tonsillectomy)
- Comparison: Compared with no treatment for the condition
- Outcome: Resolution of OME, improvement of ORCHL, or reduction of acute otitis media episodes

### Data extraction and quality assessment

For each included article, we extracted the number of CLP patients treated, patient’s age and sex, type of clefts included, clinical indications to adenoidectomy, type of clinical evaluation (for inclusion and/or outcomes), type of outcome(s) studied, and outcome(s).

Selected studies were assessed for both quality and methodological bias according to the National Heart, Lung, and Blood Institute Study Quality Assessment Tools (NHI-SQAT) [14]. Articles were rated in duplicate by two authors (A. M. S. and C.R.) and disagreements were resolved by consensus. Items were rated as good if they fulfilled at least 80% of the items required by the NHI-SQAT, fair if they fulfilled between 50 and 80% of the items, and poor if they fulfilled less than 50% of the items, respectively.

Also, the level of evidence was scored according to the Oxford Centre for Evidence-based Medicine (OCEBM) level of evidence guide [15].

### Results

### Search results

The number of unique items retrieved from each database is available in Online Resource 1. Among the 321 unique research items initially identified, a total of 18 articles were selected for full-text evaluation and further 7 were retrieved from citations and reviews. Among the 25 articles undergoing full-text evaluation, 3 studies were retained for further analysis (see Online Resource 2 for the selection process details and Online Resource 3 for details on the articles excluded during the full-text evaluation).

Two articles were case series [16, 17] and one was a retrospective cohort study [18], all published from the mid-Sixties to the early Seventies. The resulting levels of evidence according to the OCEBM scale were rated 4 for two studies and 3 for the remaining one. According to the NHI-SQAT, all articles were judged of fair quality. Most articles lacked ample information to support the comparability of patients. No significant bias emerged from the evaluation of the articles. The pooled population from the three studies was 136 patients. Table 1 reports the characteristics of the included studies, their demographics, and the type of cleft included. Sex distribution among the samples was not reported in any of the articles.

**Table 1:** Characteristics of the included studies

	Study type	OCEBM rating	NHI-SQAT rating	Number of patients	Mean age
Chalat, 1965 [16]	case series	4	F	38	N/A
Loeb, 1964 [17]	case series	4	F	15	N/A
Severeid, 1972 [18]	retrospective cohort study	3	F	83	11

*OCEBM* Oxford Centre for Evidence-Based Medicine, *NI-SQAT* National Heart, Lung, and Blood Institute Study Quality Assessment Tools, *F* fair; *N/A* not available

Table 2 reports data in terms of procedures performed (adenoidectomy or adenotonsillectomy), indications to surgery, diagnostic and outcome assessment methods, and outcomes.

**Table 2:** Data on cleft types, performed procedures, patients diagnosis, evaluation tools, and outcomes

	Type of cleft (s)	Procedure performed	Indications to surgery	Evaluation tools	Outcome(s)
Chalat, 1965 [16]	Cleft lip and/or palate	A + T	ORCHL (38 patients), RAOM (35 patients)	Clinical evaluation and PTA	Average ORCHL improved from 12.7 dB to 3.4 dB; resolution of RAOM in 28/35 patients
Loeb, 1964 [17]	Cleft palate	A or A + T	RAOM, worsening ORCHL	Clinical evaluation and PTA	Improved hearing and speech in 10 patients; hypernasality improved in 3 patients, unaltered in 12 patients
Severeid, 1972 [18]	Cleft palate	A	OME	clinical evaluation and myringotomy	OME resolution in 51/83 patients

*A* adenoidectomy, *T* = tonsillectomy, *A + T* adenotonsillectomy, *ORCHL* OME-related conductive hearing losses, *OME* otitis media with effusion, *RAOM* recurrent acute otitis media, *PTA* pure tone audiometry

In one study, all patients underwent adenotonsillectomy, in another, all patients underwent isolated adenoidectomy and, in a third study, patients underwent either adenoidectomy or adenotonsillectomy. Two out of three studies included patients with RAOM or ORCHL and one exclusively included patients with OME. The diagnosis was otoscopy-based in all studies, but employed also audiometry for the two studies treating patients with ORCHL. In the single study on OME, the diagnosis was confirmed with myringotomy in all but two patients. No study employed tympanograms. The outcomes were positive in all three articles in terms of improvement of ORCHL [16, 17], reduction of acute otitis media episodes [16, 17], and OME resolution [18], though this last result was not statistically significant when compared to no treatment in a control group of cleft palate children. Besides primary outcomes, two papers [16, 17] reported positive outcomes in subjective perceptual speech. A single study [17] reported outcomes in terms of hypernasality, which was improved in 3 out of 15 patients and unchanged in the remaining 12 patients. The author hypothesized that the removal of adenoid improved palatal muscle mobility, thus allowing for this unexpected reduction of hypernasality. The speech evaluation was performed either via an informal unspecific qualitative interview with parents and speech therapist [16] or via a non-further-specified speech therapist evaluation for hypernasality and general speech quality [17].

Finally, no reviewed study performed concurrent tympanostomy prior to or concurrent with adenoidectomy. A single study [18] performed tympanostomy in a number between 81 and 83 patients, but the timing of the procedure with regards to adenoidectomy was unclear.

Due to the heterogeneity and paucity of data, a meta-analysis could not be performed.

### Discussion

A major striking datum is derived from this systematic review: despite promising preliminary results for adenoidectomy in treating middle ear disease in the CP ± L population, research in this field seems to have stopped in the mid-Seventies. This happened despite all articles included in this review report adenoidectomy (either with or without tonsillectomy) as a valuable tool in treating middle ear disease in this population. A single article [18] failed to achieve statistical significance in its positive results and suggested age as a major confounder for the results in this population. Recent studies have indeed confirmed that that middle ear disease in CP ± L children tends to improve with age [5].

It might be objected that the articles included in the systematic review lack a prospective design and their methodology—unremarkable in their historic context—might not hold up to today’s technological standards. Nevertheless, upon rating and review, they all appear to have been conducted meticulously, and their content cannot be ignored.

Studies on the role of adenoidectomy in this population have been hampered by the constant fear that the procedure could have detrimental effects on the velopharyngeal function, often already impaired in this patient group [19]. This relatively common sequela of adenoidectomy has been linked to specific morphological characteristics [20, 21], with a globally heterogeneous prevalence across studies. It has to be noted that even such a low incidence of velopharyngeal insufficiency (VPI) in the selected studies appears too optimistic not to be related to a methodological bias in reporting complications. Such a hypothesis becomes even more realistic if we take a closer look at the speech evaluation methods used in the reviewed articles and at the scattered data reporting, as already described in the results.

The risk of VPI complicating adenoidectomy in CP ± L children led over time to a gradual abandonment of blind adenoidectomy procedures. Performing endoscopy- (and power-) assisted selective peri-tubal adenoidal resections has, in turn, become the gold standard in this patient group [22]. This change became reality as endoscopy-assisted adenoidectomy turned into a reliable tool in the general pediatric population in the 90 s. Endoscopic evaluation of the surgical field allows for selective adenoidectomy and has been widely demonstrated as a safe and reproducible tool also in the CP ± L population with no detrimental effect on speech [23] and VPI [13, 24].

Different techniques have been proposed, spanning from trans-oral suction diathermy or curettage to trans-nasal cold or powered resection [25]. Partial lateral adenoidectomy is the first endoscopic approach presented in the literature. It was nevertheless shown to predispose the patient to postoperative VPI in case of posterior pharyngeal wall anatomical irregularity leading to inadequate velopharyngeal sealing [26]. To overcome the issue, both Finkelstein et al. [26] and Stern et al. [27] suggested performing a targeted inferior or superior endoscopic partial adenoidectomy, to reduce adenoidal volume while preserving the tissue behind the soft palate. Both studies used a 0° nasal endoscope, which granted full operative control and was reported to significantly reduce postoperative complications.

Endoscopic visualization of the nasopharynx can be also obtained via oral access, as showed in several studies [22, 23] in which a superior partial adenoidectomy is performed with cold or powered instruments with the aid of a transoral 70° endoscope. Abdel-Aziz et al. [22] claim that the transoral approach enables the surgeon to efficiently inspect the velopharyngeal valve during adenoidectomy, allowing to assess which portion of the adenoidal tissue contributes the most to velopharyngeal closure. These claims have been more recently indirectly confirmed in another study [28]. In this study, Ferreira and colleagues compare adenoidectomy techniques in non-cleft children reporting that, while blind curette adenoidectomy has the same efficacy and complication rate of the endoscopic and represents a faster and cheaper alternative, endoscopy can significantly increase surgical precision.

Another study, from Askar and Quiriba [25], further underlines the pivotal role of surgical precision in cleft children’s surgery. They propose a routine coupling of nasal endoscopy with power-assisted tools to remove adenoidal tissue. In this study, while only 30% of adenoidal tissue was removed, after a 48-month follow-up, all patients had their nasal breathing difficulties solved and no case of VPI was reported. Indeed, prospective studies endoscopically comparing cases operated with curettes or power-assisted instruments [29] showed that conventional curettage adenoidectomy is less precise than endoscopic assisted adenoidectomy, especially in the choanal and tubal regions.

These studies regardless of the specific technique presented highlight the fundamental role of endoscopic assistance in treating CP ± L patients. The only reason for endoscopic to be absent from our review focused on middle ear disease in cleft children is that the only papers found in our systematic review are from the ‘70 s, i.e., well before endoscopy became an established surgical tool.

It is also to be noted that current scientific reports confirm that adenoidectomy still represents a treatment choice in CP ± L, despite its indications being presently limited to nasal breathing difficulties and obstructive sleep apnea [30, 31].

Therefore, we take into account the following:

- The preliminary good results on middle ear disease reported in the original, albeit outdated works on adenoidectomy in cleft children
- The introduction of less invasive modern endoscopic partial adenoidectomy techniques
- The efficacy of adenoidectomy in treating OME also in large-scale meta-analysis, and
- The routine use of adenoidectomy in the cleft population of other indications

It comes as a surprise that no prospective studies on this subject have been proposed. The extremely wide use of tympanostomy as a first-line treatment for OME and ORCHL in this patient group represents a further direct consequence of the paucity of data on adenoidectomy and middle ear disease in the cleft population.

It has to be noted that this systematic review is limited in its strength as it included all article types, focusing on a wide range of middle ear conditions and with heterogeneous evaluation tools, but the lack of a significant bulk of literature on the subject made any further refinement impossible. Nevertheless, a call for stronger evidence on the subject emerges preponderantly. An unclear aspect of this review is worth examining in-depth, i.e., the relationship in the CP ± L population between tympanostomy and adenoidectomy. Unfortunately, only one reviewed study reported performing tympanostomy in nearly all patients, but with unclear timing. On the other hand, tympanostomy wasn’t apparently performed in the reviewed studies. This overall management clashes with current trends in CP ± L patients with middle ear disease, so the results in these regards should be further put into context with future studies. Our literature review furthermore showed a complete lack of evidence in the use of tympanostomy tubes concurrent with adenoidectomy in the CP ± L population, as no study addressing this particular subgroup was identified.

## Conclusion

In the present context of middle ear disease in the cleft population, it would be unreasonable to suggest adenoidectomy as an alternative to tympanostomy. However, there is a population with a known risk of long-term otologic sequelae [5] in which adenoidectomy could represent a powerful additional tool that requires urgent investigation—cleft patients requiring re-tympanostomy. Prospective randomized controlled trials of partial adenoidectomy in these patients would be feasible, ethical, and might hold great potential. Possible positive results might therefore help delineate a new and wider role for this old-fashioned technique.

## Supplementary Information

Below is the link to the electronic supplementary material.

Supplementary file1 (PDF 209 kb)

Supplementary file2 (PDF 204 kb)

Supplementary file3 (PDF 233 kb)

## Abbreviations

CP ± L: Cleft palate with or without cleft lip
OME: Otitis media with effusion
RAOM: Recurrent acute otitis media
ORCHL: OME-related conductive hearing loss
PRISMA: Preferred reporting items for systematic reviews and meta-analyses
OCEBM: Oxford centre for evidence-based medicine
NHI-SQAT: National heart, lung, and blood institute study quality assessment tools
