# Supplementary material for: Adenoidectomy for middle ear disease in cleft palate children: a systematic review
Source: Eur Arch Otorhinolaryngol. 2021 Aug 28;279(3):1175–80. doi: 10.1007/s00405-021-07035-6 (PMC8897369; doi:10.1007/s00405-021-07035-6)
Supplement: Supplementary file 1 — Supplementary file1 (PDF 209 kb) [file 405_2021_7035_MOESM1_ESM.pdf]

ADENOIDECTOMY FOR MIDDLE EAR DISEASE IN CLEFT PALATE CHILDREN: A SYSTEMATIC REVIEW

Journal: European Archives of Oto-Rhino-Laryngology

Authors: Cecilia Rosso, Antonio Bulfamante, Pipolo Carlotta, Fuccillo Emanuela, Maccari Alberto, Lozza Paolo, Scotti Alberto, Pisani Antonia, Castellani Luca, De Donato Giuseppe, Tavilla Maria Chiara, Portaleone Sara, Felisati Giovanni, Saibene Alberto Maria

Correspondence to: Alberto Maria Saibene, Otolaryngology Unit - ASST Santi Paolo e Carlo. Via Antonio di Rudinì, 8 - 20142 - Milan, Italy. Phone: +39 02 8184 4249. Fax: +39 02 5032 3166. Mail: alberto.saibene@gmail.com

Online resource 1: Search strategy ad results for all consulted databases

Database	Search strategy	Date of search	Unique items found
MEDLINE	("adenoidectomy" OR "adenotonsillectomy") AND "cleft"	November 18th, 2020	161
Embase			259
Web of Science			120
Cochrane library			6
Clinicaltrials.gov			11
