# Supplementary material for: Adenoidectomy for middle ear disease in cleft palate children: a systematic review
Source: Eur Arch Otorhinolaryngol. 2021 Aug 28;279(3):1175–80. doi: 10.1007/s00405-021-07035-6 (PMC8897369; doi:10.1007/s00405-021-07035-6)

ADENOIDECTOMY FOR MIDDLE EAR DISEASE IN CLEFT PALATE CHILDREN: A SYSTEMATIC REVIEW

Journal: European Archives of Oto-Rhino-Laryngology

Authors: Cecilia Rosso, Antonio Bulfamante, Pipolo Carlotta, Fuccillo Emanuela, Maccari Alberto, Lozza Paolo, Scotti Alberto, Pisani Antonia, Castellani Luca, De Donato Giuseppe, Tavilla Maria Chiara, Portaleone Sara, Felisati Giovanni, Saibene Alberto Maria

Online resource 2: PRISMA style flow diagram of studies selection through the review

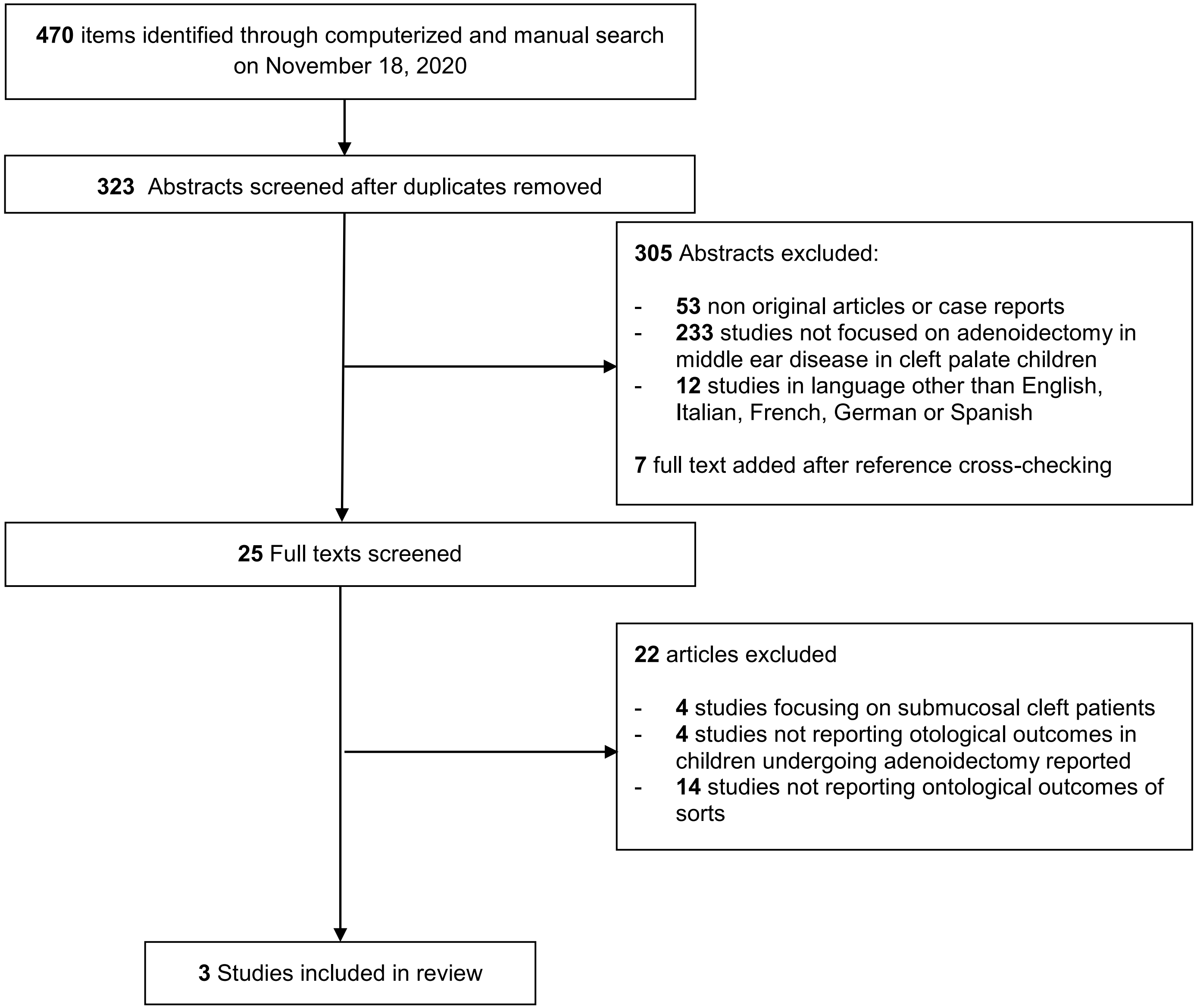

Supplement: Supplementary file 2 — Supplementary file2 (PDF 204 kb) [file 405_2021_7035_MOESM2_ESM.pdf]
