# Supplementary material for: Adenoidectomy for middle ear disease in cleft palate children: a systematic review
Source: Eur Arch Otorhinolaryngol. 2021 Aug 28;279(3):1175–80. doi: 10.1007/s00405-021-07035-6 (PMC8897369; doi:10.1007/s00405-021-07035-6)
Supplement: Supplementary file 3 — Supplementary file3 (PDF 233 kb) [file 405_2021_7035_MOESM3_ESM.pdf]

**ADENOIDECTOMY FOR MIDDLE EAR DISEASE IN CLEFT PALATE CHILDREN: A SYSTEMATIC REVIEW**

**Journal: European Archives of Oto-Rhino-Laryngology**

**Authors: Cecilia Rosso, Antonio Bulfamante, Pipolo Carlotta, Fuccillo Emanuela, Maccari Alberto, Lozza Paolo, Scotti Alberto, Pisani Antonia, Castellani Luca, De Donato Giuseppe, Tavilla Maria Chiara, Portaleone Sara, Felisati Giovanni, Saibene Alberto Maria**

**Online resource 3: List of articles excluded during the full-text evaluation**

| Full Citation | Reason for exclusion |
| --- | --- |
| Panis R. [Adenotonsillectomy in children with cleft palate. Indication, procedure, and results of 20 operations]. Laryngol Rhinol Otol (Stuttg). 1980 Feb;59(2):83-7. | No otological outcomes reported |
| Reiter R, Haase S, Brosch S. [Submucous cleft palate--an often late diagnosed malformation].Laryngorhinootologie. 2010 Jan;89(1):29-33. | Article focused only on submucosal cleft patients |
| Andreassen ML, Leeper HA, MacRae DL, Nicholson IR. Aerodynamic, acoustic, and perceptual changes following adenoidectomy. Cleft Palate Craniofac J. 1994 Jul;31(4):263-70. | No otological outcomes reported |
| Pulkkinen J, Ranta R, Heliovaara A, Haapanen ML. Craniofacial characteristics and velopharyngeal function in cleft lip/palate children with and without adenoidectomy. Eur Arch Otorhinolaryngol. 2002 Feb;259(2):100-4. | No otological outcomes reported |
| Stern Y, Segal K, Yaniv E. Endoscopic adenoidectomy in children with submucosal cleft palate. Int J Pediatr Otorhinolaryngol. 2006 Nov;70(11):1871-4. | Article focused only on submucosal cleft patients |
| Finkelstein Y, Wexler DB, Nachmani A, Ophir D. Endoscopic partial adenoidectomy for children with submucous cleft palate. Cleft Palate Craniofac J. 2002 Sep;39(5):479-86. | Article focused only on submucosal cleft patients |
| Croft CB, Shprintzen RJ, Ruben RJ. Hypernasal speech following adenotonsillectomy. Otolaryngol Head Neck Surg. 1981 Mar-Apr;89(2):179-88. | Article focused only on submucosal cleft or non-cleft patients |
| Whittemore KR Jr, Dargie JM, Dornan BK, Boudreau B. Otolaryngology Service Usage in Children | No otological outcomes reported |

| | |
|---|---|
| With Cleft Palate. Cleft Palate Craniofac J. 2018 May;55(5):743-746. | |
| Tweedie DJ, Skilbeck CJ, Wyatt ME, Cochrane LA. Partial adenoidectomy by suction diathermy in children with cleft palate, to avoid velopharyngeal insufficiency. Int J Pediatr Otorhinolaryngol. 2009 Nov;73(11):1594-7. | No otological outcomes reported |
| Askar SM, Quriba AS. Powered instrumentation for transnasal endoscopic partial adenoidectomy in children with submucosal cleft palate. Int J Pediatr Otorhinolaryngol. 2014 Feb;78(2):317-22. | No otological outcomes reported |
| Perkins JA, Sie K, Gray S. Presence of 22q11 deletion in postadenoidectomy velopharyngeal insufficiency. Arch Otolaryngol Head Neck Surg. 2000 May;126(5):645-8. | No otological outcomes reported |
| Haapanen ML, Veija M, Pettay M. Speech outcome in cleft palate patients with simultaneous primary palatal repair and adenoidectomy. Acta Otolaryngol. 1993 Jul;113(4):560-2. | No otological outcomes reported |
| Abyholm FE. Submucous cleft palate. Scand J Plast Reconstr Surg. 1976;10(3):209-12. | No otological outcomes reported |
| Pickrell KL, Massengill R Jr, Quinn G, Brooks R, Robinson M. The effect of adenoidectomy on velopharyngeal competence in cleft palate patients. Br J Plast Surg. 1976 Apr;29(2):134-6. | No otological outcomes reported |
| Drettner, B. The nasal airway and hearing in patients with cleft palate. Acta Otolaryngol. 1960 Aug;52:131-42. | No otological outcomes reported |
| Abdel-Aziz M, Khalifa B, Shawky A, Rashed M, Naguib N, Abdel-Hameed A. Trans-oral endoscopic partial adenoidectomy does not worsen the speech after cleft palate repair. Braz J Otorhinolaryngol. 2016 Jul-Aug;82(4):422-6. | No otological outcomes reported |
| Chaco, J., Yules, R.B. Velopharyngeal incompetence post tonsillo-adenoidectomy: An electromyographic study. Acta Otolaryngol. 1969 Sep;68(3):276-8. | No otological outcomes reported |
| Eufinger H, Eggeling V, Immenkamp E. Velopharyngoplasty with or without tonsillectomy and/or adenotomy--a retrospective evaluation of speech characteristics in 143 patients. J Craniomaxillofac Surg. 1994 Feb;22(1):37-42. | No otological outcomes reported |
| Sataloff J., Fraser M. Hearing loss in children with cleft palates. AMA Arch Otolaryngol. 1952;55(1):61-64. | No otological outcomes in children undergoing adenoidectomy reported |
| Halfond M., Ballenger J. An Audiologic and Otorhinologic Study of Cleft-Lip and Cleft-Palate Cases I. Audiologic Evaluation. AMA Arch Otolaryngol. 1956;64(1):58-62. | No otological outcomes in children undergoing adenoidectomy reported |
| Halfond M., Ballenger J. An Audiologic and Otorhinologic Study of Cleft-Lip and Cleft-Palate | No otological outcomes in |

| | |
|---|---|
| CasesII. Otorhinologic Evaluation. AMA Arch Otolaryngol. 1956;64(4):335-340. | children undergoing adenoidectomy reported |
| Wishart DE, Whaley JB, Wallace WB. Rhinology in children resume of and comments on the literature for 1956. Laryngoscope. 1957 Sep;67(9):833-57. | No otological outcomes in children undergoing adenoidectomy reported |
